# Envenomation by the Green Bush Viper *Atheris squamigera*^☆^

**DOI:** 10.1016/j.toxrep.2022.11.003

**Published:** 2022-11-09

**Authors:** Sam T. Ontiveros, Priya Srihari, Garret A. Winkler, Jake Del Rosso, Julia Sobel, Richard F. Clark, Alicia B. Minns

**Affiliations:** a Division of Medical Toxicology, Department of Emergency Medicine, MUSC, Charleston, USA; b Department of Emergency Medicine, Keck School of Medicine of USC, Los Angeles, USA; c Division of Medical Toxicology, Department of Emergency Medicine, UCSD, San Diego, USA

**Keywords:** Bush Viper, Venom, Antivenom, Antivenin

## Abstract

The Green Bush Viper, Atheris squamigera, is native to West and Central Africa and has few well reported envenomations. Bite victims experience dizziness, nausea, headache, regional lymphadenopathy, and localized edema. Most reports also detail severe effects including thrombocytopenia, coagulopathy, hemolysis, hemorrhage, or renal failure. Fatalities are reported, but poorly described. There is no specific antivenom for A. squamigera, but non-species specific antivenom has been reported helpful in several cases. We report the case of a 36-year-old woman who was bitten by a green bush viper and was treated with several non-species specific antivenoms. There were no complications to antivenom administration and the patient experienced a milder envenomation than detailed in previous reports.

## Highlights

• The Green Bush Viper has no specific antivenom and envenomation is poorly reported.
• Local and systemic effects can be seen. Multi-organ failure and severe bleeding reported.
• *Echis* antivenoms are reported helpful, but early administration has not previously been reported.

## Graphical Abstract

**Figure:**
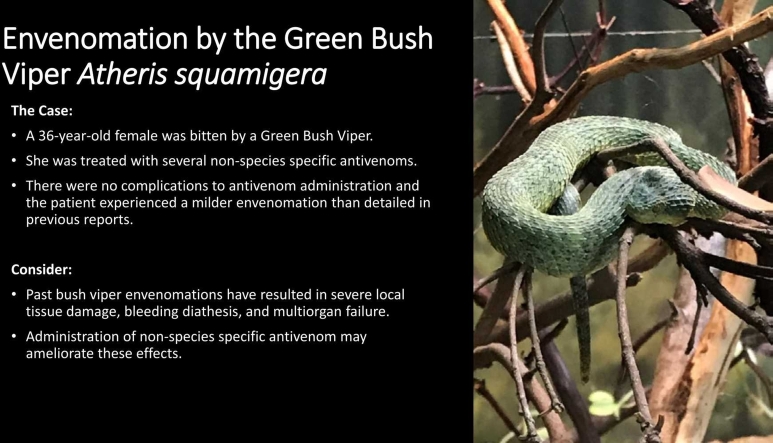

## 1 Introduction

Bush vipers are a small group of African vipers comprising the *Atheris* genus [1], [2]. The Green Bush Viper, *Atheris squamigera*, is found in forested regions of West and Central Africa. Envenomations are reported, but few detailed accounts exist. Bite victims experience dizziness, nausea, headache, regional lymphadenopathy, and localized edema. Severe envenomation may include thrombocytopenia, coagulopathy, hemolysis, hemorrhage, and renal failure. Fatalities are reported, but poorly described [1], [2], [5]. There is no specific antivenom for *A. squamigera*, and it remains unclear if antivenom derived from closely related vipers is helpful [2], [3], [4], [5]. We report a case of bush viper envenomation treated with antivenoms from alternative Old and New World vipers.

## 2 Case

A 36-year-old female zookeeper presented to the emergency department within an hour of being bitten in the right hand by a green bush viper while cleaning enclosures. She reported immediate pain, bleeding, and progressive swelling with associated nausea and headache. Vital signs were heart rate of 72 beats per minute (bpm), blood pressure of 132/78 mmHg, respiratory rate of 17 breaths per minute, and oxygen saturation of 99% on room air. Examination revealed bleeding from two punctures on the right dorsal hand with surrounding edema and induration. Bleeding stopped within 10 min, but the edema and ecchymosis progressed proximally from the wrist at roughly 2 cm per 30 min. The hand had brisk capillary refill and was neurovascularly intact. Initial laboratory investigations were a white blood cell count of 6.7k/mm^3^, hemoglobin of 12.2 g/dL, platelets of 196k/mm^3^, PT of 10.9, PTT of 25, INR of 1.0, fibrinogen of 180 mg/dL, D-Dimer of 224 ng/ml, and creatinine kinase of 320 U/L. A metabolic panel and hepatic transaminases were normal. Four vials of Inoserp Pan-Africa® F(ab’)_2_ were infused intravenously in doses of two vials at 1.5 h and 2.25 h post-bite without control of symptoms. Four additional vials were administered 3 h after the bite, but the patient continued to have pain, progression of swelling, and declining platelets and fibrinogen. Ten vials of Antivipmyn TRI® were administered 5.5 h after the initial bite. Edema and ecchymosis progressed to include the entire upper extremity, axilla, and chest with worsening regional lymphadenopathy, pain, paresthesias, malaise, and nausea. Platelets and fibrinogen continued to drop. Ten vials of ANAVIP® were administered 8.5 h post-bite with no further symptom progression. By the next morning, the patient’s fibrinogen and platelet count were improving and her hemoglobin remained stable. No blood products were administered. On hospital day 3 the patient’s edema began decreasing and she was discharged. Routine follow up labs on day 7 were normal (Table 1).

**Table 1:** Timeline of Patient’s Hospital Course.

Time After Envenomation (hh:mm)	Hemoglobin (g/dL)	Platelet (x10^3^/mm^3^)	Fibrinogen (mg/dL)
00:43	12.2	196	180
02:19	13.1	168	156
03:47	12.9	150	135
08:30	12.3	103	80
11:30	12.3	92	76
16:43	12.4	97	91
21:09	12.5	93	115
27:32	12.2	90	125
33:25	12.2	92	152
39:30	12.6	93	183

The patient was administered Inoserp Pan-Africa F(ab)2 2 vials at 1.5 hrs, 2 vials at 2.25 hrs, and 4 vials at 3 hrs post-bite. The patient was administered Antivypmen Tri 10 vials 5.5 hrs hours post-bite. The patient was administered Anavip 10 vials 8.5 h post-bite.

## 3 Discussion

Bush viper envenomation is poorly reported and the optimal management remains unknown. Given the severity of symptoms and fatalities seen in previous reports, our patient was administered available antivenoms to try to moderate these effects [2], [3], [4], [5]. Four zoo provided foreign antivenoms were considered. A whole IgG product was discarded given the reported high risk of hypersensitivity reactions, leaving two F(ab’)_2_ products. *Echis* species are closely related to *Atheris* and an antivenom containing *E. leucogaster* and *E. ocellatus* (FAV Afrique) was previously reported helpful in treating a severe *Atheris* envenomation [3], [4]. Analysis of *E. ocellatus* venom also showed similar peptide sequences to *A. squamigera* [3]. We chose to administer Inoserp Pan-Africa® as it contained all the named species in FAV Afrique®. Eight total vials were given in doses of two vials, but local swelling continued to progress with decreasing platelets and fibrinogen. Our early administration of *Echis* antivenom differs from previous “successful” reports of antivenom use. However, previous cases administered antivenom late in the clinical course and improvements in those cases may have represented the natural course of envenomation. The lack of cross-reactivity between genera would not be surprising, given cross reactivity of *Echis* antivenoms even between *Echis* species is limited [6].

While there are no reports of new world viper antivenoms effectively treating *Atheris* envenomation, the local availability, excellent safety profile, and concern for severe envenomation prompted our administration of two additional antivenoms. Ten vials of zoo provided Antivipmyn TRI® did not halt symptom progression and, in a last attempt to avoid the bleeding diathesis, hemolysis, and multi-organ dysfunction seen in previous cases, ten vials of ANAVIP® were administered before shifting entirely to supportive management. While local symptom progression halted and the coagulopathy improved, it is unclear whether this was due to antivenom administration. It is reasonable to assume not all bush viper envenomations are as severe as previously reported cases. Since our patient was treated, a subsequent mild envenomation was reported in Florida where antivenom was initially ordered, but not given [7].

## 4 Conclusion

Our case resulted in a less severe clinical course when compared to prior reported *Atheris* species envenomations, but does not conclusively demonstrate the effectiveness of non-specific antivenoms. While it is possible that antivenom administration moderated the observed clinical effects, it is also possible that our findings simply represent the natural course of disease in a milder case of envenomation. Further work is needed to determine the optimal treatment of *Atheris* envenomation.

## Declaration of Competing Interest

The authors declare that they have no known competing financial interests or personal relationships that could have appeared to influence the work reported in this paper.

## Data Availability

The data that has been used is confidential.

## References

[1] Mebs D., Holada K., Kornalík F., Simák J., Vanková H., Müller D., Schoenemann H., Lange H., Herrmann H.W. (1998). Severe coagulopathy after a bite of a green bush viper (Atheris squamiger): case report and biochemical analysis of the venom. Toxicon.

[2] Hatten B.W., Bueso A., French L.K., Hendrickson R.G., Horowitz B.Z. (2013). Envenomation by the Great Lakes Bush Viper (Atheris nitschei). Clin. Toxicol..

[3] Wang H., Chen X., König E., Zhou M., Wang L., Chen T., Shaw C. (2018). Comparative profiling of three atheris snake venoms: A. squamigera, A. nitschei and A. chlorechis. Protein J..

[4] Top L.J., Tulleken J.E., Ligtenberg J.J., Meertens J.H., van der Werf T.S., Zijlstra J.G. (2006). Serious envenomation after a snakebite by a Western bush viper (Atheris chlorechis) in the Netherlands: a case report. Neth. J. Med..

[5] Branch W.R., Haagner G.V., Morgan D.R., Lanoie L.O. (1991). Venoms and snakebite. J. Herpetol. Assoc. Afr..

[6] Rogalski A., Soerensen C., Op den Brouw B., Lister C., Dashevsky D., Arbuckle K., Gloria A., Zdenek C.N., Casewell N.R., Gutiérrez J.M., Wüster W., Ali S.A., Masci P., Rowley P., Frank N., Fry B.G. (2017). Differential procoagulant effects of saw-scaled viper (Serpentes: Viperidae: Echis) snake venoms on human plasma and the narrow taxonomic ranges of antivenom efficacies. Toxicol. Lett..

[7] Imperato N.S., Amaducci A.M., Abo B.N. (2022). African bush viper envenomation: a case report. Cureus.

