# Supplementary material for: Envenomation by the Green Bush Viper Atheris squamigera
Source: Toxicol Rep. 2022 Nov 9;9:2018–9. doi: 10.1016/j.toxrep.2022.11.003 (PMC9764243; doi:10.1016/j.toxrep.2022.11.003)
Supplement: Supplementary file 1 — Supplementary material [file mmc1.pdf]

NO. OF ANIMALS
IN CAGE

3

African Bush Viper
Atheris sp.
Antivenom- None/Envelope #3
Immobolize & Sling

African Bush Viper
Atheris sp.
Antivenom- None/Envelope #3
Immobolize & Sling

African Bush Viper
Atheris sp.
Antivenom- None/Envelope #3
Immobolize & Sling
