# Supplementary figures and images for: Envenomation by the Green Bush Viper Atheris squamigera
Source: Toxicol Rep. 2022 Nov 9;9:2018–9. doi: 10.1016/j.toxrep.2022.11.003 (PMC9764243; doi:10.1016/j.toxrep.2022.11.003)

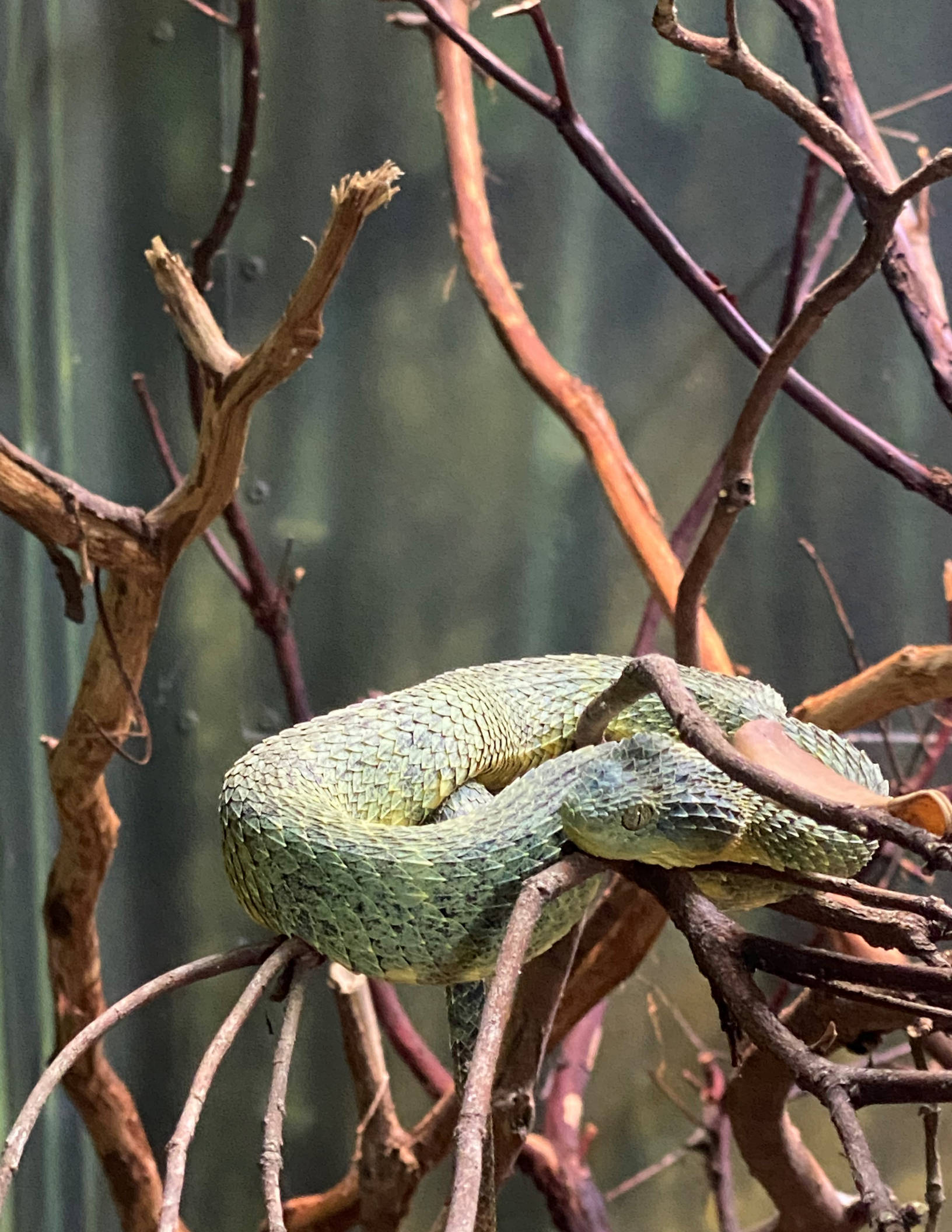

Supplement: Supplementary file 2 — Supplementary material [file mmc2.pdf]
